# Neuromuscular ultrasound as a biomarker in the SOD1 mouse model of amyotrophic lateral sclerosis

**DOI:** 10.1371/journal.pone.0353397

**Published:** 2026-07-14

**Authors:** Camilla Wohnrade, Nadine Thau-Habermann, Thomas Gschwendtberger, Julia Rückoldt, Zhong Huang, Stefanie Schreiber, Kirsten Haastert-Talini, Susanne Petri

**Affiliations:** 1 Department of Neurology and Clinical Neurophysiology, Hannover Medical School, Hannover, Lower Saxony, Germany; 2 Institute of Neuroanatomy and Cell Biology, Hannover Medical School, Hannover, Lower Saxony, Germany; 3 Department of Neurology, Otto-von-Guericke-University Magdeburg, Magdeburg, Saxony-Anhalt, Germany; 4 Center for Systems Neuroscience (ZSN) Hannover, Hannover, Lower Saxony, Germany; University of Minnesota Medical School, UNITED STATES OF AMERICA

## Abstract

A progression marker that indicates early disease-related changes and treatment responses in the to date incurable neurodegenerative disease amyotrophic lateral sclerosis (ALS) is highly desirable. Translation of therapeutics that have been successful in *in vivo* models into trials in human patients has proven difficult in recent decades. This failure can be attributed, at least in part, to the lack of specific biomarkers for ALS diagnosis and progression in human ALS patients as well as in *in vivo* models. Neuromuscular ultrasound is an easily accessible, non-invasive tool to support diagnosis of ALS in humans. Our current study shows for the first time that the disease can be detected in an ALS mouse model with the help of neuromuscular ultrasound. We characterized disease progression regarding changes in the peripheral nerves and muscles of the hind limb in the SOD1^G93A^ mouse model of ALS using different techniques (neuromuscular ultrasound, electroneurography, motor function tests, phenotypic assessments and histology). By neuromuscular ultrasound, we measured the cross-sectional area and diameter of the sciatic nerve and analyzed hind limb muscle texture and thickness. Our results show that motor neuron loss and muscle atrophy – analogous to ALS in humans – can be measured by ultrasound in the SOD1^G93A^ mouse model. Changes in nerve and muscle morphology appear at the same time or even before changes in the established tests (including electroneurographic measurements) performed *in vivo* in this model. Correlations with histologic features of disease progression make neuromuscular ultrasound a sensitive, non-invasive outcome marker for preclinical studies.

## Introduction

Amyotrophic lateral sclerosis (ALS) is the most common, adult-onset motor neuron disorder. Clinical manifestations include muscle weakness and atrophy, dysarthria, dysphagia, weight loss and respiratory insufficiency, leading to death within 3–5 years after onset [1,2].

To date, disease-modifying treatment options are limited: Riluzole, a glutamate antagonist, has shown modest efficacy, while the benefits of edaravone, a free radical scavenger approved in a few countries, have not been confirmed in a phase 3 study [3–5]. Recently, promising treatment strategies have emerged for familial ALS with mutations in the SOD1 (superoxide dismutase 1), C9orf72 and FUS (fused in sarcoma) genes (NCT03626012, NCT04768972) [6,7], though they are suitable for up to 10% of ALS patients only. For sporadic ALS, more than 50 drugs with different mechanisms of action have shown promising results in preclinical studies in *in vivo* models over the past decades, but translation into clinical trials is prone to fail [8–10]. These negative results might reflect clinical and pathophysiological heterogeneity of the disease as well as the lack of biomarkers for adequate quantification of disease progression and treatment response. While neurofilaments have moved into focus as “fluid” diagnostic and prognostic biomarkers for ALS [11–13], disease progression is routinely monitored using manual strength testing, the ALS functional rating scale and the King´s staging [14–16].

High frequency diagnostic ultrasound is a readily accessible, non-invasive modality to assess structural changes in peripheral nerves and muscles and in this application is referred to as neuromuscular ultrasound. In ALS, neuromuscular ultrasound is an established method for diagnosis and assessment of disease progression: It can detect fasciculations – as early signs of lower motor neuron impairment – with higher sensitivity than electromyography [17,18]. Further, quantity of striated muscles in the extremities (measured in terms of thickness) is reduced and decreases throughout the disease course [19–26]. The decline in muscle thickness correlates with ALSFRS-R [27]. Echo intensity as measure of muscle quality is increased in correlation with muscle strength and alterations precede clinically manifest paresis [22–24,28–30]. Ultrasound of peripheral nerves has shown cervical nerve root atrophy as well as proximal atrophy of median and ulnar nerves with reduced cross-sectional area and increased distal-proximal ratio for the latter [23,31–35]. Although not routinely applied, neuromuscular ultrasound thus offers optimal properties for the monitoring of disease progression.

Regarding preclinical studies in *in vivo* models of ALS, biomarkers used for quantification of disease progression comprise weight loss, strength testing, gait analysis and investigator specific neurological scoring systems [9]. The first and best-characterized animal model of ALS is the mutant SOD1^G93A^ mouse, which overexpresses mutant human SOD1 and develops adult-onset motor neuron disease similar to the human disease. Different pathological mechanisms converge to motor neuron loss in the spinal cord followed by muscle atrophy and weakness in hind – and forelimbs [10,36,37]. Disease progression most commonly is quantified using weight loss, scores for the severity of paralysis, rotarod test, hanging wire, step length and run time. Nerve conduction studies and motor unit number estimation (MUNE) are also applied [38–43]. All of these techniques have limitations, including operator variability, lack of sensitivity and invasiveness with the need of anesthesia or euthanasia. Further, they do not translate directly to the human disease, where staging is based on more nuanced functional scores.

In the present study, we aimed to establish neuromuscular ultrasound as biomarker of disease stages in the mutant SOD1^G93A^ mouse model of ALS. We performed ultrasound of the sciatic nerve and limb muscles at three presymptomatic and three symptomatic time points during the disease course and set the findings in the context of well-established biomarkers such as motor performance testing, weight, nerve conduction studies as well as histological hallmarks of the disease. We successfully defined a new, sensitive, non-invasive biomarker that applies to the human disease and the mouse model, and assessed nerve and muscle morphometry *in vivo*.

## Materials and methods

### Animals

Transgenic SOD1^G93A^ mice (B6.Cg-Tg(SOD1*G93A)1Gur/J; RRID:IMSR_JAX:004435) overexpressing the human *SOD1* mutation [36] were obtained from The Jackson Laboratory (Bar Harbor, ME, USA). By mating transgenic males with wild-type (C57BL/6J; RRID:IMSR_JAX:000664) females, transgenic hemizygous mice were bred and genotyped by polymerase chain reaction (PCR). Wild-type littermates were used as control group. Mice were kept under controlled conditions (20–24°C, 45–65% humidity, 14h/10h light/dark cycle) and had free access to food and water. Groups of up to five animals of the same sex were kept in Makrolon cages type II L (UNO, Zevenaar, Netherlands). All procedures were carried out in accordance with internationally accepted principles in the care and use of experimental animals. The experiments were in accordance with the German Animal Welfare Legislation and approved by the local Institutional Animal Care and Research Advisory Committee and permitted by the Lower Saxony State Office for Consumer Protection and Food Safety (reference numbers 18/3016 and 19/3072).

Transgenic SOD1^G93A^ animals were compared with their wild-type siblings at six different time points (aged 7, 10, 13, 16, 19 and 22 weeks). Animals were trained for motor tests for one week at each time point. This was followed by examinations/analyses, always in the following order: 1. motor tests, 2. ultrasound examinations, 3. electroneurography, and 4. euthanasia and tissue sampling for histological examinations (S1 Fig).

### Ultrasound techniques

We used the Vevo® 2100 Imaging system (Visual Sonics Inc., Toronto, Canada) with a 40 MHz (22–55 MHz) real time linear array transducer to visualize the sciatic nerve and hind limb muscles. It offers spacial resolution down to 30 µm. Imaging was performed with the transducer fixed with the equipment provided by Visual Sonics. System-settings such as frequency (40 MHz), gain (15 dB), depth (8 mm for longitudinal plane, 5 mm for axial plane), time gain compensation and focus were kept unchanged. All experiments were carried out by the same investigators, who were blinded to the genotype of the animals. Though, blinding was only possible up to week 19, because of visible phenotypic changes in the animals thereafter. The same applies to the motor testing and nerve conduction studies.

The measurements were carried out under isoflurane inhalation anesthesia and the right hind leg was depilated. Eye ointment was applied before the measurements to protect the cornea. Hereafter, animals were positioned prone with hind legs stretched at a 45 degree angle relative to the spine. Feet were fixed with the plantar side on a heating panel. The sciatic notch was palpated and the location was marked on the skin as point of reference. To improve acoustic coupling, ultrasound gel was applied on the skin. First, the right sciatic nerve was displayed in the longitudinal plane by positioning the transducer perpendicular and parallel to the thigh: Proximal, the lumbosacral portion of the spine and the hip were visualized, distal, the cartilage portion of the knee and the proximal part of the gastrocnemius muscle were included in the image. The sciatic nerve was identified by anatomical landmarks and typical morphology (hyperechoic rim with hypoechoic honeycomb structured inner portion). Several images were taken to maximize thickness/diameter of nerve and muscle. Further, the thigh was displayed in the axial plane so that a cross-sectional view of the sciatic nerve was obtained. The sciatic nerve was visualized from the trochanter major to where the cranial portion of the gastrocnemius muscle is perforated. Right before perforation the sciatic nerve separates into tibial and peroneal nerves, which run alongside each other for several more millimeters. Images were obtained distal right after the bifurcation, at the median portion of the thigh and proximal where the sciatic nerve passes by the hip.

PW (pulsed wave) Doppler Mode was used in longitudinal as well as transversal planes to distinguish between blood vessels and nerve. To differentiate between tendons/fascia and nerve, the transducer was tilted slightly sideways, as tendons change their echotexture when viewed in a different angle whereas nerves do not. Generally, the pressure applied to the transducer was minimized to avoid muscle/ nerve compression and distorted measurements. Also, oblique scanning was avoided, as it might increase the echotexture and size of the muscle/nerve.

### Image processing

The resulting images had a resolution of 1176 x 800 Pixels (Pixel spacing 0,013750 0,013752) (scale: 72,73 Px/mm) with 256 Grey levels (8-bit) and were stored as “.DCM” files without compression or losses. Primary image processing and analysis were performed by the same investigator using open access ImageJ (v 1.52a) software. In longitudinal planes, maximal nerve diameter was measured setting the measurement points just inside the hyperechoic rim at two positions: approximately 0.5–1 mm proximal to the beginning of the gastrocnemius muscle (distal diameter) and at the position where the nerve winds around the hip (proximal diameter). Muscle thickness was determined as opening angle of the gastrocnemius muscle using the same image and the “angle tool”. The vertex was positioned at the distal sciatic nerve; the legs of the angle were adjusted to the hyperechoic fascia of the muscle.

Cross sectional area (CSA) of the sciatic nerve was measured in the axial plane using the tool “freehand selection” and tracing just inside the hyperechoic rim of the nerve, corresponding to the epineurium (see [35]). Measurements were taken proximal, where the sciatic nerve runs around the hip. As the nerve occasionally had indistinct borders due to acoustic shadows, measurement of the CSA was performed three times per animal in three different pictures and all three measurements were averaged to obtain a mean CSA. Thickness of the biceps femoris muscle was determined in the distal axial plane placing the measurement points on the inside of the hyperechoic fascia surrounding the muscle. As point of reference the bifurcation into tibial and peroneal nerve of the sciatic nerve was used. Next, echo intensity of the muscle was measured in a standard manner using grey-scale analysis: A rectangular region of interest (ROI) of 2.00 x 0.80 mm was selected manually, that included a homogenous proportion of the biceps femoris muscle and excluded any fascia or acoustic shadowing. Using the “histogram” tool, a histogram displaying the frequency of grey values (from 0 = black to 255 = white) was generated and the mean grey level was recorded as echo intensity [29,44–47].

### Phenotype and motor assessment

General condition was assessed weekly as previously described [38,41] using a scoring system ranging from 5 to 1 (score of 5 = without any symptoms, 4 = destabilized gait/first signs of paralysis, 3 = obvious paralysis of hind limbs, 2 = crawling on forelimbs only and 1 = lying on the side/not able to turn within 5 sec.). When animals reached a score of 1 or lost more than 20% of their initial weight, they were euthanized. From day 105 and during the experiments, the general condition was assessed daily. Body weight was recorded weekly using a normal digital balance.

Motor function was assessed using rotarod, hanging wire and footprint analysis as previously described [38,41]. After an adaption period of five days of daily training, motor function was tested prior to any other procedures. Briefly, a rotarod apparatus from IITC (IITC Life Science Inc., Calif., USA) was used with an initial speed of 1 rpm. Within 180 s rotation speed increased to 180 rpm and the time until the animals fell from the rotating cylinder was registered (max. 180 s). Footprints and runtime were obtained by painting hind limbs of the mice with black ink and letting them run along a 50 cm gangway. The time the animals needed to cover the distance was documented. Step length was measured using open access ImageJ Software (v.1.52a). To measure the grip strength, the mice were placed upside down on a grid surrounded by an impassable frame and the time the animals were able to hold on to the grid was measured (maximum 120 s). The scores given for the motor tests, sonography and electroneurography were averaged for the analysis.

### Electroneurography

For electroneurographic analyses, animals were anesthetized using inhalation anesthesia (isoflurane) and the left hind leg was depilated. Prior to the electrophysiological measurements Carprofen (0.01 ml/100g; 5 mg/kg; Rimadyl®, Pfizer GmbH, Karlsruhe, Germany) was administrated subcutaneously for analgesia, and dexpanthenol eye ointment (Bepanthen® Augen- und Nasensalbe, Bayer Vital GmbH, Leverkusen, Germany) was applied to the eyes to prevent corneal dehydration. Body temperature was kept constant between 34–36°C by placing the mice on a thermostated heating pad (direct current). The portable EMG device (Natus Keypoint Focus, Natus Europe GmbH, Planegg, Germany) together with the computer program Keypoint.net (version 2.32) was used for the measurements. For motor nerve conduction studies, the sciatic nerve was stimulated percutaneously with single pulses of 0.1 ms and 1 Hz placed through a pair of needle electrodes (Spes Medica monopolar disposable needle electrode, 13 mm x 33G, GVB geliMED KG, Bad Segeberg, Germany) placed proximal at the sciatic notch and distal at the popliteal fossa as described before [48]. The distance between the two stimulation electrodes was measured. Reference electrodes were placed subcutaneously at a distance of a few millimeters from the stimulation electrodes. The maximum compound muscle action potential (CMAP) after supramaximal stimulation was recorded with needle electrodes placed in the gastrocnemius, tibialis anterior or plantaris (plantar interossei) muscles. The reference electrode for all three muscles was placed in the fourth toe. The ground electrode was placed subcutaneously rostral to the stimulation electrodes. Amplitude (baseline to peak, mean proximal/distal) and latency (time from stimulus to onset of first negative deflection, mean proximal/distal) were measured. The nerve conduction velocity (NCV) was calculated using the latency differences of the proximal and distal stimulation and the stimulation distance (mean NCV per gastrocnemius/tibialis/plantaris muscle) (see [42] and [43]).

### Histomorphology

Following electroneurography, animals were euthanized and samples for histological evaluation were collected. In detail, the right sciatic nerve, right gastrocnemius muscle and lumbar part of the spinal cord were removed.

### Motor neuron survival, astrocytosis and microgliosis in the spinal cord

As previously described [49], lumbar spinal cord tissue was fixed overnight in 4% paraformaldehyde (PFA) and then cryopreserved at least overnight in 30% sucrose. After embedding in cryoprotection compound (Sakura Finetek Germany GmbH, Staufen, Germany), the tissue was stored at −80°C until the sections were prepared using a cryotome (10x 12 µm serial sections per slide).

Nissl staining was performed to evaluate the motor neurons. For this purpose, tissue sections were stained with 0.5% thionin, dehydrated in graded ethanols and xylene (Mallinckrodt Baker B.V., Deventer, Netherlands) and covered with Eukitt rapid mounting medium (Sigma-Aldrich, Steinheim, Germany). Ten cross sections of the spinal cord were taken at 20x magnification with an Olympus BX61 microscope. Cell software (Olympus cellSens Dimension 1.18, Hamburg, Germany) was used to evaluate the average number of motor neurons per section. Cells in the area of the ventral horn with a diameter of >200 µm² were defined as motor neurons [50]. To visualize astrocytosis and microgliosis in the lumbar spinal cord, fluorescence immunohistochemical staining with cell specific antibodies was performed after one hour of blocking (1% milk powder, 1% BSA, 0.1% TritonX100 and 0.1% Tween20 in PBS) overnight at 4°C. GFAP (1:500; DAKO Z0334) was used as astrocyte marker and Iba-1 (1:500; Wako 0019–19741) as microglia marker. Immunofluorescence was induced by the antigen-specific secondary antibody (ThermoFisher A-21428). Mowiol (Roth) was used as a covering medium. In 10 spinal cord cross-sections, 20 images per animal if possible of the ventral spinal cord per staining were taken with the Olympus BX61 microscope. The evaluation was carried out via a color contrast analysis as previously described using ImageJ (v 1.52a) (see [49]).

### Sciatic nerve histomorphology

The nerves were histomorphometrically analyzed in line with a previously reported protocol [51]. The sciatic nerve was dissected to get the samples. Karnovsky fixative (2% PFA, 2.5% glutaraldehyde in 0.2 M sodium cacodylate buffer, pH 7.3) was applied to the dissected nerve segments for 24 hours. After rinsing the samples with 0.1 M sodium cacodylate buffer supplemented with 7.5% sucrose, they were post-fixed for 1.5 hours in 1% osmium tetroxide. The myelin sheath was stained with 1% potassium dichromate (for 24 hours), 25% ethanol (for 24 hours), and hematoxylin (0.5% hematoxylin in 25% ethanol, for 24 hours) [52]. Following that, the samples were embedded in Epon and sliced into 1 µm thick semi-thin cross-sections. Toluidine blue staining was used to increase the coloration of the myelin sheaths. The cross-sections were analyzed with a BX50 microscope (Olympus Europa SE & Co. KG, Germany), a prior controller (MBF Bioscience, USA), and Stereo Investigator software, version 11.04. (MBF Bioscience, USA).

Stereological evaluations were conducted by an investigator blinded to the origin and genotype of the samples in accordance with previous reports [53–56]. In brief, two randomly chosen sections of each sample were included in the following analysis for all axons in the nerve cross-sections, including those that were positioned outside the fascicles’ perineurium but still within the nerves’ epineurium. For calculating the total number of myelinated fibers (in 100x magnification) and the respective nerve fiber density with a two-dimensional dissector (optical fractionator; grid size: 150 × 150 µm; counting frame size: 30 × 30 µm), the cross-sectional area was determined (in 10x magnification). Also the “fiber tips” were counted as suggested by others [57].

Sciatic nerve samples from all time points were further analyzed for the appearance of normal fibers (healthy and regularly myelinated axons) in comparison to abnormal ones, e.g., degenerating axons. In order to make the estimation more accurate with regard to the various types of abnormal nerve fibers in the whole sample, the counting frame size was adjusted to 40 × 40 µm and the grid size was adjusted to 120 × 120 µm. The following categories were defined for this type of analysis, normal fiber (with clear axonal area and well-wrapped myelin sheaths) and abnormal fibers. Abnormal fibers were further divided in three sub-categories depending on the location of the abnormality at: 1) the internal aspect (e.g., inner layers of the myelin sheath or axon disintegration), 2) the external aspect (e.g., outer layers of the myelin sheath), or 3) both = completely degenerated fibers with no clear myelin profile and axonal disintegration).

Four view fields were recorded for each section (eight in total) of each sample at a magnification of 100x for analyzing the nerve morphometry. To determine axon diameter, fiber diameter, myelin thickness, and the related g-ratio, ImageJ (version 1.48; National Institutes of Health, USA) was expanded with a *g*-ratio plug-in [53,54,58]. The results were collected assuming that the axons had a circular form. Axons with full degeneration or an indistinguishable myelin boundary were eliminated from morphometric analysis.

### Neuromuscular junction (NMJ) immunostaining

For endplate (NMJ) analysis, gastrocnemius muscle tissue was harvested and fixed overnight in 4% PFA and then cryopreserved at least overnight in 30% sucrose. After embedding in cryoprotection compound, the tissue was stored at −80°C until further processing. A series of 20 µm longitudinal sections of the muscle were prepared using a cryotome (3 slides with 6 sections each). Before staining the NMJ, the free aldehydes were blocked with 0.1 M glycine solution (in PBS) for 30 minutes. Before overnight incubation with the labeled α-bungarotoxin (BTX; 2 µg/ml in blocking solution; CF®555 (stock 0.5 mg/ml) Biotium #00018), blocking was performed for one hour with 2% milk powder and 0.5% TritonX100 in PBS. The next day, sections were incubated in 1 mM fresh cupric sulphate (CuSO4) solution (in 50 mM ammonium acetate; pH 5.0). After mounting with Kaiser’s glycerol gelatin (Roth), images were taken with an Olympus BX61 microscope. For the area/size determination of the NMJ, 20 plane endplates per sample were captured. The NMJs were recorded in 8-bit type images using the ImageJ program. The Polygon tool was used to outline the endplates. The area was determined using color threshold analysis. Six sections were counted under the microscope to determine the number of NMJs per section.

### Statistics

Statistical analysis was performed using GraphPad Prism version 10.3.0 (Graph Pad Software Inc., USA). For motor assessment, ultrasound, electrophysiological and histological data, significant differences between wild-type and mutant SOD1^G93A^ mice in each age cohort were determined using an ordinary two-way ANOVA and Bonferroni’s multiple comparisons test, with a single pooled variance. Bivariate linear correlations were calculated using the Pearson correlation coefficient. Receiver operating characteristic (ROC) curves were generated to explore the performance of CSA of the proximal and diameter of the of the distal sciatic nerve and echo intensity of the biceps femoris muscle to distinguish between wild-type and transgenic animals. The area under the (ROC) curve (AUC) was composed and Youden’s index highest value was used to determine the optimal cut-off for CSA and echo intensity as well as sensitivity and specificity. As the cut-offs were generated and tested in the same data set, ROC analysis remains exploratory.

## Results

### Changes in the sciatic nerve and muscles of the hind limbs in mutant SOD1^G93A^ mice can be visualized successfully using Neuromuscular ultrasound

**Fig 1:**
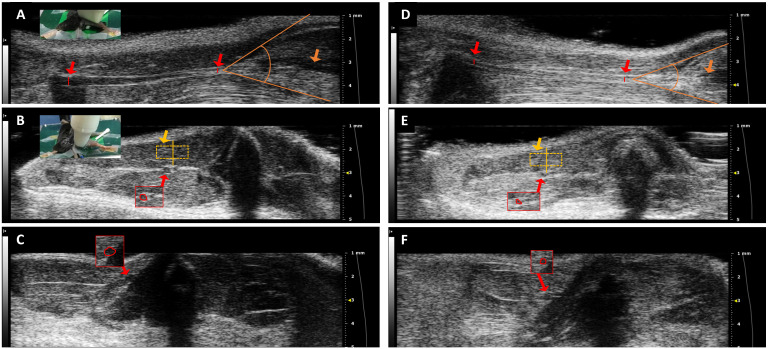
Exemplary sonographic images of the hind limb. (A, B, C) wild-type and (D, E, F) transgenic week 19: (A/D) longitudinal section (B/E, C/F) transverse section. The sciatic nerve and its measurement sites are marked with red arrows. The diameter was measured in the longitudinal plane (A/D) at a proximal site (left red arrow) where the nerve winds around the hip and at a distal site (right red arrow) at the beginning of the gastrocnemius muscle. The opening angle of the gastrocnemius muscle was estimated by positioning the vertex on the distal sciatic nerve and by adjusting the legs to the hyperechoic muscle fascia (A/D). The sciatic nerve cross-sectional area (CSA) was measured in the transverse sections, distally at the level of the knee (B/E) and proximally next to the hip (C/F) as shown. Echo intensity and thickness of the biceps femoris muscle (yellow arrow) were analyzed in a distal transverse section (B/E). The echo intensity was analyzed in a defined rectangular ROI of 2.00 x 0.80 mm and the thickness was determined in the axial plane as indicated.

Neuromuscular ultrasound was well tolerated by all animals; no side effects other than the effects associated with anesthesia or depilation of the hind leg occurred. After awakening all animals recovered and showed no new motor impairments especially of the examined hind limb. Visualization of the hind limb soft tissue including sciatic nerve and muscles was feasible, easy to learn and well replicable. Fig 1 presents an exemplary ultrasound image in the longitudinal (Fig 1A/D) and transversal plane (Fig 1B/E and 1C/F). Anatomical variations were infrequent, mostly comprising a more proximal bifurcation of the sciatic nerve into tibial and peroneal nerves on the lower thigh level. No fasciculations were detected during evaluation of muscle tissue.

In the longitudinal plane, the proximal and distal diameter of the sciatic nerve and the opening angle of the gastrocnemius muscle, as substitute for the thickness of the muscle, were measured (as indicated in Fig 1A/D). The proximal and distal nerve cross-sectional area (CSA), as well as the thickness and the echo intensity of the biceps femoris muscle were determined in corresponding cross-sectional images of the hind limb (as indicated in Fig 1B/E, 1C/F).

### Evaluation of nerve morphology shows early decrease in nerve cross-sectional area and diameter in mutant SOD1^G93A^ mice

**Fig 2:**
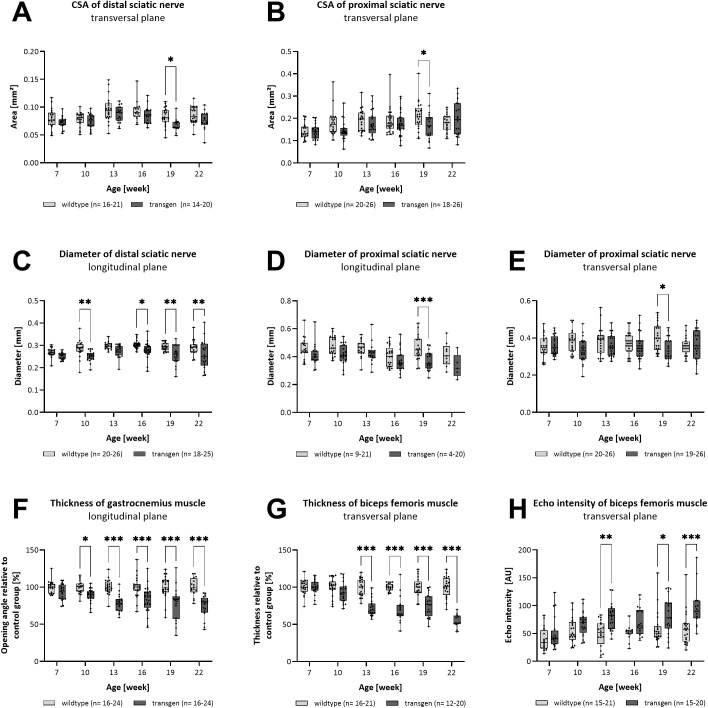
Results of the quantitative ultrasound analysis of the right sciatic nerve and hind limb muscles. **(A, B)** For the evaluation of the nerve cross-sectional area (CSA), a strong interindividual scatter was found. Proximally **(B)**, however, the CSA of the mutant SOD1^G93A^ mice was significantly smaller compared to the wild-type animals in week 19. This significant difference can also be seen when analyzing the proximal diameter of the nerve **(D, E)**. It should be noted that in the cohorts aged 10, 13, 16, 19 and 22 weeks, the distal diameter of the sciatic nerve was significantly smaller in the mutant SOD1^G93A^ mice compared to the wild-type animals **(C)**. **(F-H)** The analyses of the gastrocnemius muscle and the biceps femoris muscle show a lower thickness and higher echo intensity in the mutant SOD1^G93A^ mice cohorts aged 10, 13, 16, 19 and 22 weeks. Graphs are depicted as interleaved box & whiskers from min. to max. (all data points are shown; the n value is given in the graph). Statistical analysis was performed by using a two-way ANOVA followed by Bonferroni`s multiple comparisons test. “*” indicates *p* < 0.05; “**” indicates *p* < 0.01; “***” indicates *p* < 0.001.

The diameter and cross-sectional area of the distal sciatic nerve varied widely within each age group and were difficult to measure due to their size of < 0.1 mm² and the measurement inaccuracy of the freehand selection tool used (Fig 2A). The same was observed for the CSA of the proximal sciatic nerve, most likely due to difficulties in visualisation in the proximity of cartilaginous structures. However, we were able to measure significant differences in cross-sectional area (Fig 2B). The diameter of the distal sciatic nerve measured in the longitudinal plane was significantly smaller in transgenic animals compared to wild-type animals in the cohorts aged 10, 13, 16, 19 and 22 weeks (Fig 2C). The diameter (Fig 2D and 2E) of the proximal sciatic nerve differed significantly between wild-type and mutant SOD1^G93A^ mice in week 19. The diameter or cross-sectional area of the sciatic nerve did not change significantly when comparing younger and older animals. Sonography results at various time points corresponding to different disease stages are shown in S2 and S3 Figs. Body weight of the animals did not correlate with the data collected by sonography once animals were fully grown (S4 Fig). ROC analysis showed limited suitability for CSA of the proximal sciatic nerve to discriminate between wild-type and transgenic animals. Only at 19 weeks, AUC approached 0.7 (AUC 0.68, p = 0.034, CI 0.51–0.85) with an optimal cut-off of 0.172 mm² (sensitivity 63%, specificity 71%, N = 40). In contrast, the diameter of the distal sciatic nerve revealed an AUC ≥ 0.7 to distinguish wild-type from transgenic animals starting from 7 weeks onwards. The optimal cut-off ranged between 0.26 and 0.29 mm (Table 1).

**Table 1:** ROC analysis of the suitability of the diameter of the distal sciatic nerve to distinguish between wild-type and transgenic animals.

Age	N	Area under the curve (AUC)	Optimal cut-off (mm)	Sensitivity	Specificity
7 weeks	40	0.79***	0.26	80%	65%
10 weeks	40	0.83***	0.27	80%	85%
13 weeks	40	0.77***	0.28	65%	80%
16 weeks	51	0.79***	0.29	68%	89%
19 weeks	40	0.77**	0.27	68%	91%
22 weeks	38	0.69*	0.26	56%	90%

Youden’s index highest value was calculated to determine the optimal cut-off as well as sensitivity and specificity. “*” indicates *p* < 0.05; “**” indicates *p* < 0.01; “***” indicates *p* < 0.001.

### Evaluation of muscle morphology shows a decrease in thickness and an increase in echo intensity in mutant SOD1^G93A^ mice

The opening angle of the gastrocnemius muscle was significantly smaller in the mutant SOD1^G93A^ mice compared to wild-types in the matched cohorts aged 10, 13, 16, 19 and 22 weeks (Fig 2F). The thickness of the biceps femoris muscle was significantly decreased in the mutant SOD1^G93A^ mice compared to controls for the cohorts aged 13, 16, 19 and 22 weeks (Fig 2G). Also, the echo intensity of the biceps femoris muscle was significantly increased in the mutant SOD1^G93A^ mice cohorts aged 13, 16, 19 and 22 weeks, while a trend can already be observed in the younger animals (Fig 2H). Even though the thickness of the biceps femoris muscle and echo intensity tended to increase with age in wild-type animals, there were no significant differences between the age cohorts. ROC analysis of echo intensity of the biceps femoris muscle revealed an AUC > 0.7 to distinguish wild-type from transgenic animals starting from 13 weeks onwards. The optimal cut-off ranged between 58.2 and 83.5 AU (Table 2).

**Table 2:** ROC analysis of the suitability of echo intensity of the biceps femoris muscle to distinguish between wild-type and transgenic animals.

Age	N	Area under the curve (AUC)	Optimal cut-off (AU)	Sensitivity	Specificity
7 weeks	40	0.63 (ns)	nc	nc	nc
10 weeks	40	0.68*	60.5	65%	65%
13 weeks	40	0.78***	83.5	45%	100%
16 weeks	33	0.73*	60.0	93%	61%
19 weeks	40	0.77***	58.2	79%	76%
22 weeks	35	0.84***	72.8	80%	80%

Youden’s index highest value was calculated to determine the optimal cut-off as well as sensitivity and specificity. “*” indicates *p* < 0.05; “**” indicates *p* < 0.01; “***” indicates *p* < 0.001.

### Phenotypic changes in mutant SOD1^G93A^ mice occur at the same time or after changes detectable by neuromuscular ultrasound

**Fig 3:**
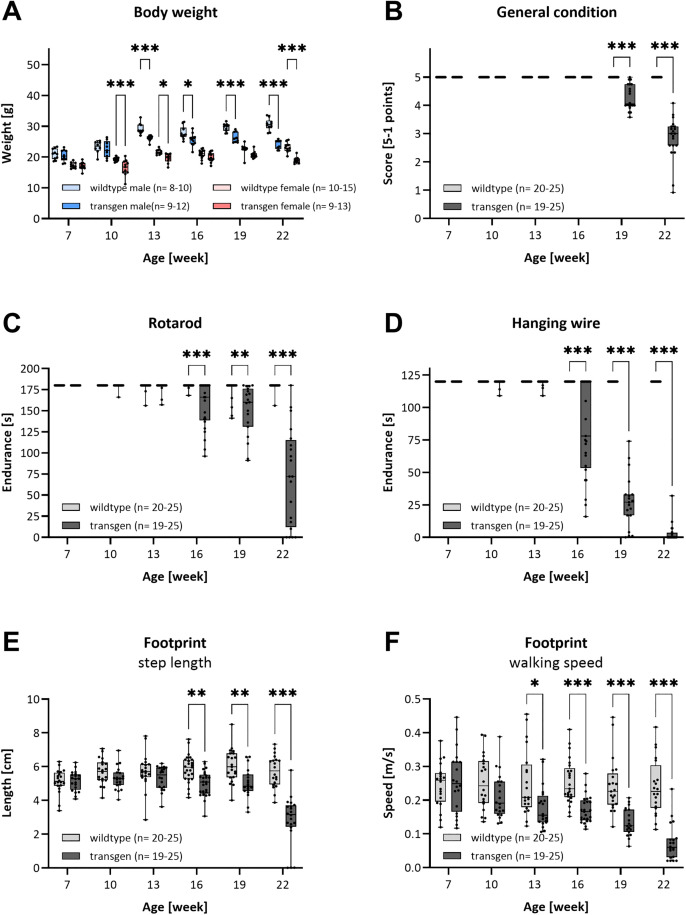
Results of the phenotypic and motor assessments. Body weight decreased earliest in the mutant SOD1^G93A^ mice **(A)**, followed by walking speed **(F)**, endurance in motor strength and coordination testing and step length **(C-E)**. Visible gait abnormalities and paralysis of the hind limbs occurred first at the age of 19 weeks **(B)**. Graphs are depicted as interleaved box & whiskers from min. to max. (All data points are shown; the n value is given in the graph.). Statistical analysis was performed by using two-way ANOVA followed by Bonferroni`s multiple comparisons test. “*” indicates *p* < 0.05; “**” indicates *p* < 0.01; “***” indicates *p* < 0.001.

Mutant SOD1^G93A^ mice cohorts showed a significant decrease in body weight starting from week ten compared to age-matched wild-type mice (Fig 3A). General condition deteriorated notably in mutant SOD1^G93A^ mice starting at the age of 19 weeks, when the first animals showed gait abnormalities and paralyses of the hind legs (Fig 3B). In motor assessments via Rotarod (Fig 3C), hanging wire (Fig 3D) and step length (Fig 3E) analyses, mutant SOD1^G93A^ mice performed significantly worse compared to control cohorts aged 16, 19 and 22 weeks. Walking speed on a 50 cm gangway was significantly slower in the cohort saged 13 weeks and older (Fig 3F).

### Differences in the electroneurography occur at the same time as the changes in the neuromuscular ultrasound

**Fig 4:**
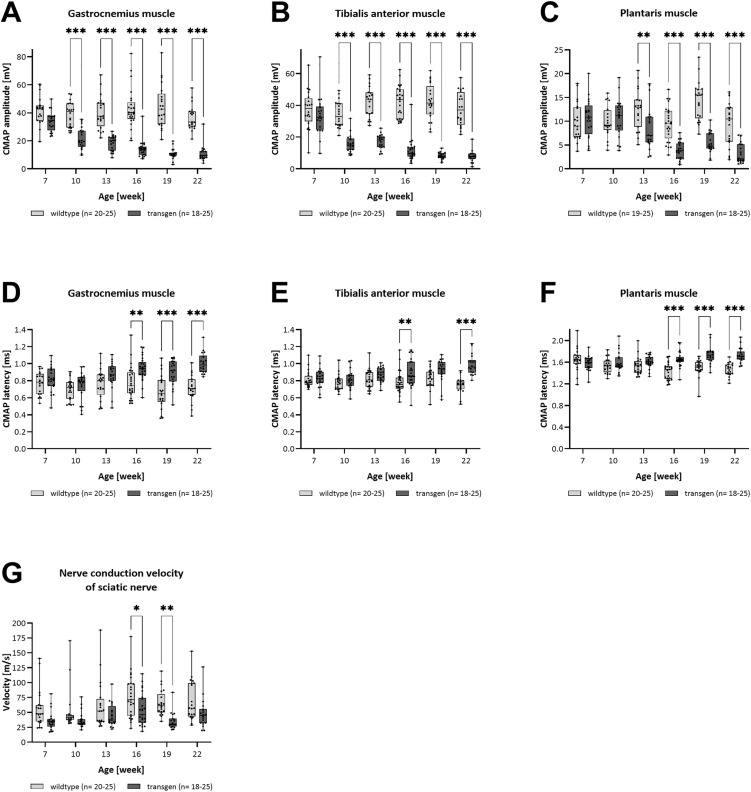
Results of nerve conduction studies. While the amplitude of the CMAP from the gastrocnemius and tibialis anterior muscles was decreased in mutant SOD1^G93A^ mice in the cohorts aged ten weeks and older **(A, B)**, the CMAP amplitude from the plantaris muscle was decreased significantly at the age of 13 weeks at the earliest **(C)**, and the distal motor latency was prolonged compared to age-matched wild-type cohorts from 16 weeks of age **(D-F)**. A reduced nerve conduction velocity (NCV) was also observed in the mutant SOD1^G93A^ mice, which became significant at 16 and 19 weeks of age **(G)**. Graphs are depicted as interleaved box & whiskers from min. to max. (All data points are shown; the n value is given in the graph.). Statistical analysis was performed by using two-way ANOVA followed by Bonferroni`s multiple comparisons test. “*” indicates *p* < 0.05; “**” indicates *p* < 0.01; “***” indicates *p* < 0.001.

The compound muscle action potential (CMAP) amplitude recorded from the gastrocnemius, tibialis anterior and plantaris muscles was significantly lower in mutant SOD1^G93A^ mice cohorts aged ten or 13 weeks and older, respectively (Fig 4A-C). Motor latency recorded in all muscles was considerably extended in mutant SOD1^G93A^ mice cohorts aged 16, 19 and 22 weeks (Fig 4D-F). Nerve conduction velocity was significantly reduced in the mutant SOD1^G93A^ mice aged 16 and 19 weeks (Fig 4G).

### Histologically significant differences between mutant SOD1^G93A^ and wild-type mice can only be detected after the changes in the ultrasound examinations

#### Spinal cord.

**Fig 5:**
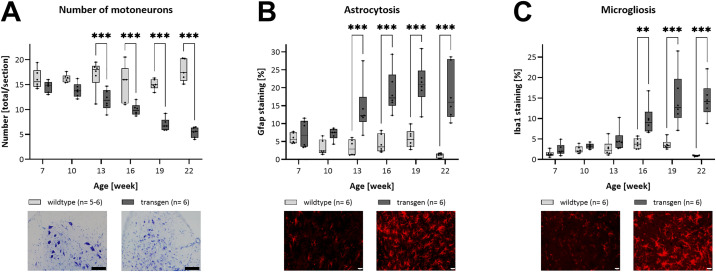
Histological quantification of motor neurons, astrocytosis and microgliosis in the spinal cord. The number of motor neurons in the lumbar section of the spinal cord significantly decreased in the mutant SOD1^G93A^ mice from the age of 13 weeks **(A)**. Increased astrocytosis **(B)** and microgliosis **(C)** became apparent at the age of 13 and 16 weeks, respectively. Exemplary images are shown for week 19 (scale bar Nissl staining: 100 µm; scale bar Gfap and Iba1 staining: 20 µm). Graphs are depicted as interleaved box & whiskers from min. to max. (All data points are shown; the respective n values are provided in the graphs.). Statistical analysis was performed by using two-way ANOVA followed by Bonferroni`s multiple comparisons test. “*” indicates *p* < 0.05; “**” indicates *p* < 0.01; “***” indicates *p* < 0.001.

Motor neuron numbers of mutant SOD1^G93A^ mice decreased with age as shown by Nissl staining (Fig 5A). Mutant SOD1^G93A^ mice cohorts had significantly fewer motor neurons compared to wild-types starting at week 13. Similarly, astrocytosis as detected by GFAP staining was increased significantly in mutant SOD1^G93A^ mice starting at week 13 (Fig 5B). Microgliosis as measured by Iba-1 immunostaining was increased in mutant SOD1^G93A^ mice beginning in week 16 (Fig 5C).

#### Sciatic nerve.

**Fig 6:**
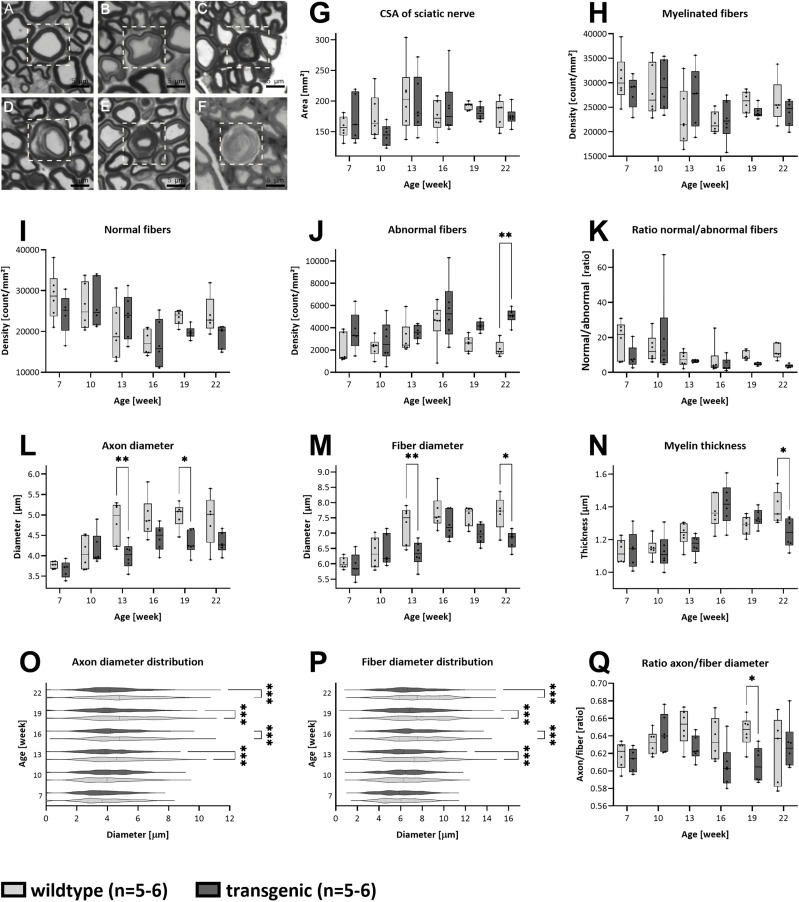
Histomorphometrical analysis of the sciatic nerve. **(A-F)** Representative photomicrographs of semi-thin, toluidine blue stained cross-sections of the nerve segments. The white dotted boxes show: **(A)** normal fiber, abnormality at the internal aspect: **(B)** inner layers of the myelin sheath or **(C)** axonal disintegration, **(D, E)** abnormality at the external aspect, **(F)** abnormal both at the internal and external aspects (completely degenerated fibers with no clear myelin profile and axonal disintegration). Scale bar = 5µm. **(G, H)** Investigation of the cross-sectional area (CSA) **(G)** and nerve fiber density (normal and abnormal myelinated fibers/mm^2^) **(H)** of the sciatic nerve at different time points. **(I-K)** Density of normal and abnormal myelinated fibers. Graphical presentation of the results derived from stereological analysis of sciatic nerve cross-sections at different time points. **(I)** Density of normal nerve fibers in each group at different time points. **(J)** Density of abnormal nerve fibers (all categories) in each group at different time points. **(K)** Ratio of fibers/mm^2^ of normal to abnormal fibers at different time points. **(L-N, Q)** Graphical presentation of the results derived from morphometrical analysis of sciatic nerve cross-sections at different time points: **(L)** Axon diameter, **(M)** Fiber diameter, **(N)** Myelin thickness, and **(Q)** Ratio axon/fiber diameter. **(O, P)** Analysis of myelinated axons **(O)** and fiber diameter distribution **(P)** at different time points. Graphs are depicted as interleaved box & whiskers from min. to max. (All data points are shown; n = 5-6). Statistical analysis was performed by using two-way ANOVA followed by Bonferroni`s multiple comparisons test. “*” indicates *p* < 0.05; “**” indicates *p* < 0.01; “***” indicates *p* < 0.001.

No significant differences were observed between the wild-type and mutant SOD1^G93A^ mice in terms of cross-sectional area (Fig 6G) or nerve fiber density (Fig 6H) in any age cohorts. Quantification of normal and abnormal fibers within the nerve cross-sections did not reveal significantly more fibers with normal aspects in the wild-type samples (Fig 6J). On the other hand, quantification of abnormal fiber aspects showed a significantly increased number in samples from mutant SOD1^G93A^ mice aged 22 weeks (Fig 6J).

Axon diameter was decreased in mutant SOD1^G93A^ mice aged 13, 16, 19 and 22 weeks compared to controls. This decrease was significant at weeks 13 and 19 (Fig 6L). The same was true for the fiber diameter. It showed a significant decrease in the 13th and 22nd week (Fig 6M). The ratio of axon to fiber diameter decreased over the with increasing age of mice the cohorts which became significant at week 19 (Fig 6Q). Both the axon (Fig 6O) and the fiber diameter (Fig 6P) distribution was shifted significantly in the cohorts aged 13, 16, 19 and 22 weeks.

### Neuromuscular junction (NMJ)

**Fig 7:**
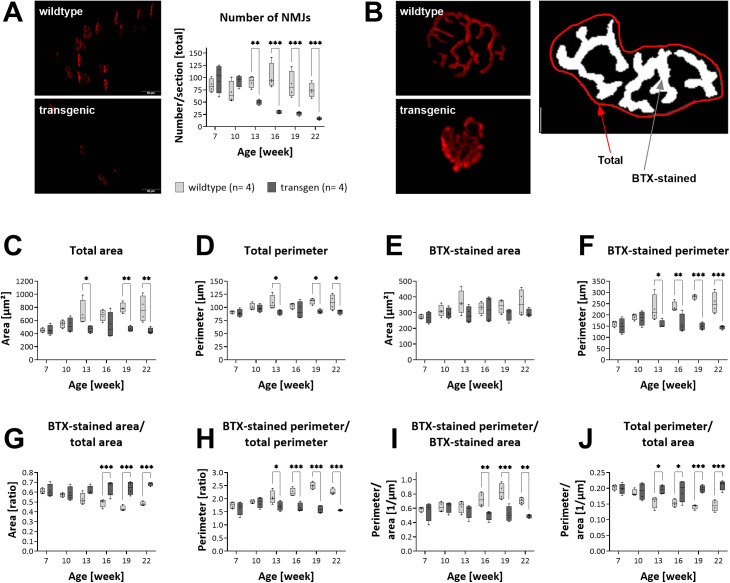
Evaluation of the neuromuscular junction (NMJ) in longitudinal sections of the gastrocnemius muscle. From week 13, the mutant SOD1^G93A^ mice not only exhibited a significantly reduced number **(A)** of NMJs in the gastrocnemius muscle, but the morphology **(C-J)** of the NMJs also changed compared to the age-matched control cohort. From week 13 onwards, there was a reduced total area **(C)** (see **(B)**) and perimeter **(D)**. The area stained with BTX **(E)** did not change, but the perimeter **(F)** of the stained area was smaller in the mutant SOD1^G93A^ mice compared to the wild-type animals. The BTX area relative to the total area **(G)** was significantly larger in the mutant SOD1^G93A^ mice cohorts from the age of 16 weeks onwards. In line with this, the ratio of the perimeter of the BTX-stained area to the total perimeter **(H)** showed significant differences from week 13 onwards. In the cohorts aged 16, 19 and 22 weeks, the ratio of the perimeter of the BTX-stained area to the BTX-stained area also differed significantly **(I)**. The perimeter of the total area relative to the total area **(J)** was larger in SOD1^G93A^ mutant mice than in wild-type mice in the cohorts aged 13 weeks and older. Graphs are depicted as interleaved box & whiskers from min. to max. (All data points are shown; the n value is given in the graph.). Statistical analysis was performed by using two-way ANOVA followed by Bonferroni`s multiple comparisons test. “*” indicates *p* < 0.05; “**” indicates *p* < 0.01; “***” indicates *p* < 0.001.

The quantification of the postsynaptic NMJs showed a decrease in the mutant SOD1^G93A^ mice cohorts from week 13 onwards (Fig 7A). Starting at the same time point, morphological changes in the postsynaptic NMJs were observed as shown in (Fig 7B): the total area (Fig 7C) covered by the post-synapse and its perimeter (Fig 7D) were significantly reduced in the mutant SOD1^G93A^ mice cohorts aged 13 weeks and older. By contrast, there was no difference between the transgenic and wild-type animals in the area stained by BTX (Fig 7E). However, the perimeter of the BTX-stained area was significantly smaller in the transgenic animals (Fig 7F). When the ratio of BTX-stained area to total area was considered, significant differences were observed in the cohorts aged 16 weeks and older (Fig 7G). When considering the ratio of the perimeter of the BTX-stained area to the total perimeter (Fig 7H), significant differences were observed in the cohorts aged 13 weeks and older, as were the ratios of the total perimeter to the perimeter of the total area (Fig 7J). From week 16 onwards, the ratio of the perimeter of the BTX-stained area to the BTX-stained area also differed significantly (Fig 7I). In the transgenic animals, the number of NMJs decreased during the course of the disease, and morphologically, there were also a decrease in branching and atrophy of the postsynaptic NMJs detectable.

### Correlation analysis between sonographic and phenotypic, electroneurographic and histological changes

**Fig 8:**
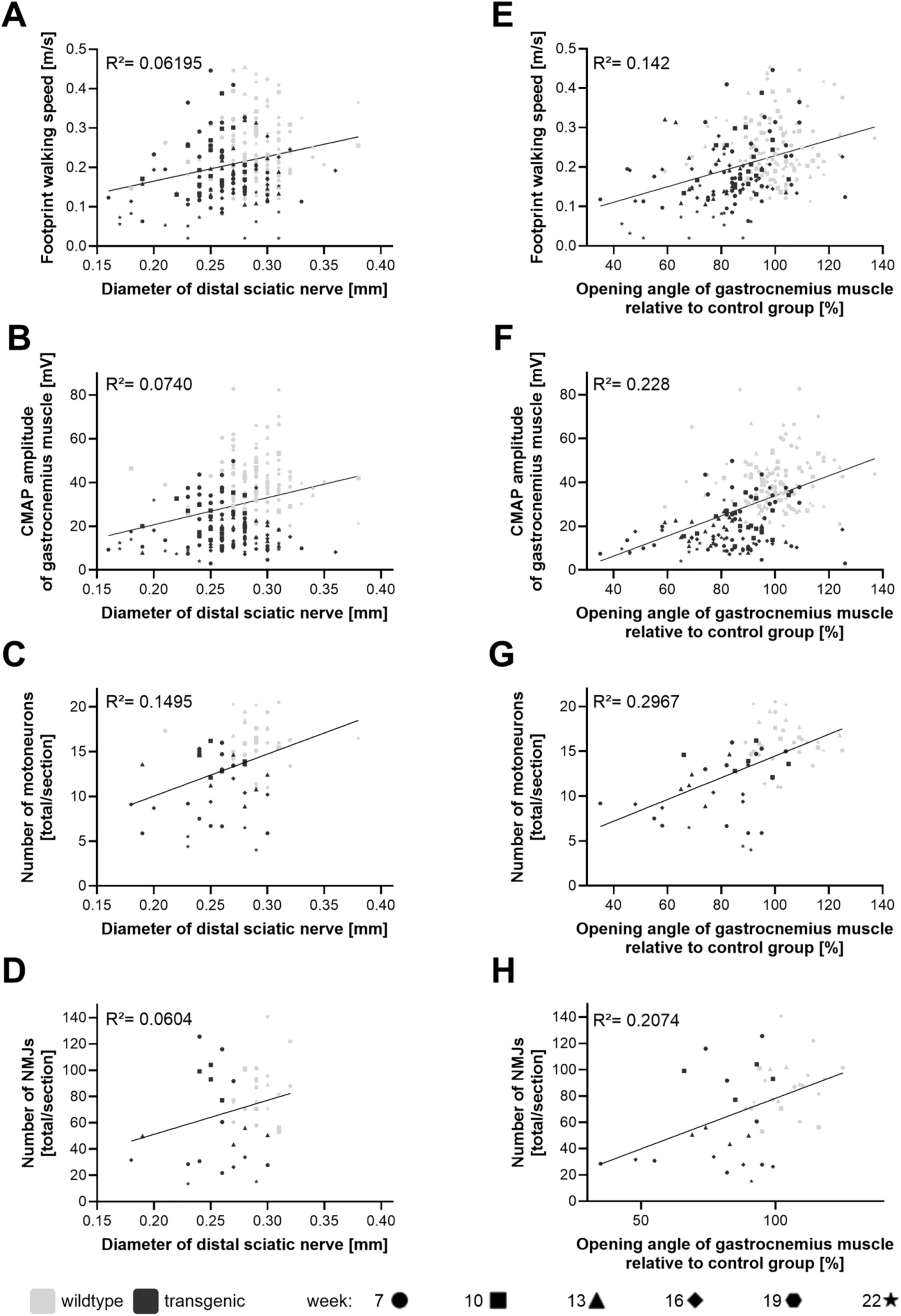
Correlation analysis of nerve and muscle ultrasound with phenotypic, electroneurographic and histological changes. The sonographically determined diameter of the distal sciatic nerve correlated significantly with walking speed (Pearson 0.2489 ***, N = 245, **A)**, CMAP amplitude of the gastrocnemius muscle (Pearson 0.272 ***, N = 244, **B)** and with the number of motor neurons (Pearson 0.3866 **, N = 68, **C)**. Correlations with the number of NMJs (Pearson 0.2458 ns, N = 46, **D)** were not significant. The opening angle of the gastrocnemius muscle correlated significantly with walking speed (Pearson 0.377 ***, N = 237, **E)** and CMAP amplitude of the gastrocnemius muscle (Pearson 0.478 ***, N = 236, **F)**, the number of motor neurons (Pearson 0.5438 ***, N = 65, **G**) and the number of NMJs (Pearson 0.4555 **, N = 43, **H)**. Graphs are depicted as scatterplot with regression line. (All data points are shown.). Statistical analysis was performed by using Pearson correlation coefficient. “*” indicates *p* < 0.05; “**” indicates *p* < 0.01; “***” indicates *p* < 0.001.

Correlation analysis revealed significant positive correlations of the diameter of the distal sciatic nerve measured by ultrasound with walking speed, CMAP amplitude of the gastrocnemius muscle and the number of motor neurons (Fig 8A, B, C). However, there was no significant correlation with the number of NMJs (Fig 8D). The opening angle of the gastrocnemius muscle correlated significantly with walking speed and CMAP amplitude of the gastrocnemius muscle, the number of motor neurons and the number of NMJs (Fig 8E - H).

## Discussion

SOD1^G93A^ mice develop a rapidly progressive motor neuron disease. Current outcome measures (weight loss, severity of paralysis, motor testing and nerve conduction studies) are only moderately satisfactory markers to assess disease progression and effects of experimental drugs. In this study, we were able to show 1. that neuromuscular ultrasound is feasible in the SOD1^G93A^ mouse model, 2. that changes in nerve and muscle morphometry are similar to the changes described in human ALS, and 3. that the ultrasonographic alterations precede or coincide with abnormalities in the standard techniques used for characterizing disease progression in this mouse model.

In human ALS, neuromuscular ultrasound is challenging because of increased echogenicity and loss of tissue heterogeneity [59]. Further, ultrasonographic parameters can vary considerably with age, sex, or muscle group, making the establishment of normal values difficult. In the SOD1^G93A^ mouse model, disease heterogeneity is minimal and restricted to controllable factors such as genetic background and sex. This makes the SOD1 mouse model suitable to explore disease mechanisms and effects of experimental drugs. So far, only few studies applied nerve and muscle ultrasound in small (rodent) animal models: In 2005 de Kool et al. were the first to perform ultrasound imaging of a peripheral nerve (peroneal nerve) in a small animal model [60]. Kuffler et al. examined the rat sciatic nerve lesion model and successfully identified anatomical detail on the location and type of damage to the sciatic nerve [61]. Nijhuis et al. performed ultrasound-guided needle positioning near the sciatic nerve for selectively eliciting CMAPs, while Kim et al. used focused ultrasound on the sciatic nerve [60–63]. Even though the murine sciatic nerve is around four times smaller than its human counterpart (0.4 mm and 2 cm in diameter, respectively), we were able to reliably visualize it in the SOD1^G93A^ mouse model and trace it from its emergence from the pelvis up to its bifurcation into peroneal and tibial nerve in the popliteal fossa. Further, it was possible to identify individual muscles as well as muscle groups in the mouse hind limb and obtain cross sectional images of the whole hind limb. Thanks to new high resolution ultrasound technology, neuromuscular ultrasound thus is available for preclinical studies with the purpose of monitoring disease progression in small rodents involving delicate anatomical structures.

Although the ultrasound technique is easy to learn, users need to operate with care and the pressure and tilt applied to the transducer need to be monitored continuously. A further skill that the user must acquire is the correct positioning of the transducer. The integration of special holding devices for the transducers can facilitate the resolution of positioning issues. Also, smaller neuroanatomical structures such as the median or ulnar nerve are too small to be reliably visualized in a mouse model. This poses practical challenges to the application of neuromuscular ultrasound in small animals.

The diameter of the distal sciatic nerve was significantly smaller in mutant SOD1^G93A^ mice compared to age-matched wild-types (Fig 2C). This finding is in line with changes described in human ALS where median nerve, ulnar nerve and cervical nerve root atrophy have been described using ultrasound [32–35]. The most likely explanation for the observed peripheral nerve atrophy is progressive motor axon loss, which is supported by the significantly smaller CMAP amplitude detected in the gastrocnemius (and tibialis anterior) muscles and the reduced number of motor neurons in the spinal cord. Sonographically detected reduced nerve diameter correlated positively with both, CMAP amplitude and number of motor neurons, and appeared as early as at the age of ten weeks in mutant SOD1^G93A^ mice cohorts.

Sonographic CSA of the distal sciatic nerve did not show a clear pattern of atrophy, which most likely traces back to the fact that it is difficult to measure due to its size of < 0.1 mm² in the mouse model and imprecise rounding of the free hand selection tool used. We did not find consistent sonographic differences in proximal sciatic nerve diameter or CSA, which might partly be attributed to technical issues, as visualization of nerve boundaries in the proximity of cartilaginous/osseous structures such as the spinal column and ilium proved challenging. Then again, it might reflect propagation of denervation in a distal to proximal direction as has been proposed within the dying-back hypothesis. The dying-back hypothesis assumes that the disease is initiated distally at the neuromuscular junction and progresses in a retro-grade fashion to affect the axons and consequently motor neuron cell bodies [64–66]. In support of this, in human ALS the disease usually presents with distal muscle weakness spreading to adjacent and more proximal muscles [67] and electrophysiological alterations are first seen in distal muscles [68]. In accordance, in our study mass and echo intensity of the biceps femoris muscle, which is innervated by a more proximal branch of the sciatic nerve, were altered several weeks later (13 weeks) compared to the gastrocnemius muscle (seven weeks). Nevertheless, as we applied different methods to determine muscle mass for gastrocnemius and biceps femoris muscles (opening angle and thickness, respectively), this remains hypothetical.

In human ALS, ultrasonographic studies have shown proximal nerve atrophy as well [23,33,34]: the wrist to forearm ratio of the ulnar nerve and distal-proximal ratio of the median nerve were described as increased, while CSA of cervical nerve roots was reduced. In our study, the CSA and diameter of the proximal sciatic nerve were significantly smaller exclusively in animals aged 19 weeks. This could indicate, that proximal nerve atrophy occurs later during the course of disease at least in this model.

Unfortunately, we were not able to confirm our ultrasonographic findings regarding peripheral nerve atrophy in the stereological histological analysis: histological CSA and nerve fiber density were not significantly altered in transgene SOD1 mutant animals. A possible explanation might be that for nerve histomorphometry, the proximal portion of the sciatic nerve was extracted, for which we did not find a significantly reduced diameter or CSA in the ultrasonographic evaluation either. However, we histologically detected an increased proportion of abnormal fibers in samples from mutant SOD1^G93A^ mice cohorts and the axon and fiber diameter was decreased at the age of 13, 16, 19 and 22 weeks. At the same time points, the number of NMJs was significantly decreased in mutant SOD1^G93A^ mice cohorts. These findings support the hypothesis, that macroscopic and microscopic abnormalities of the nerve ascend from distal to proximal, while signaling along the nerve already is impaired – this has also been described in the works of Mejia Maza et al. and Fischer et al. [64,69]. It should be noted that a limitation of our histological examinations of the neuromuscular junctions is that bungarotoxin stains only the postsynaptic endplates; it is therefore not possible to determine whether these are still fully innervated or have already been denervated, as this would require additional markers, like synaptic vesicle protein 2.

Ultrasonographic evaluation of muscles in human ALS has revealed reduced thickness and increased echo intensity, a strong correlation of abnormalities with muscle strength and abnormalities already being evident in muscles with preserved strength [27,29,35,47,70]. In line with this, we found that in the mutant SOD1^G93A^ mouse model, ultrasound can detect reduced muscle mass as early as at the age of 10 weeks, while motor deficits became apparent rather late during the disease course at the age of 13–16 weeks. Muscle mass (measured as opening angle and thickness by ultrasound) correlated positively with motor performance, CMAP amplitude derived from the gastrocnemius muscle as well as the number of motor neurons in the spinal cord and number of NMJs, indicating that muscle ultrasound is a valid parameter to measure disease severity in this animal model. Further, muscle ultrasound is easier to perform compared to nerve ultrasound and less susceptible to accuracy errors due to the size and superficial location of the muscle tissue. Muscle ultrasound might therefore function as a surrogate for nerve morphology evaluation.

**Fig 9:**
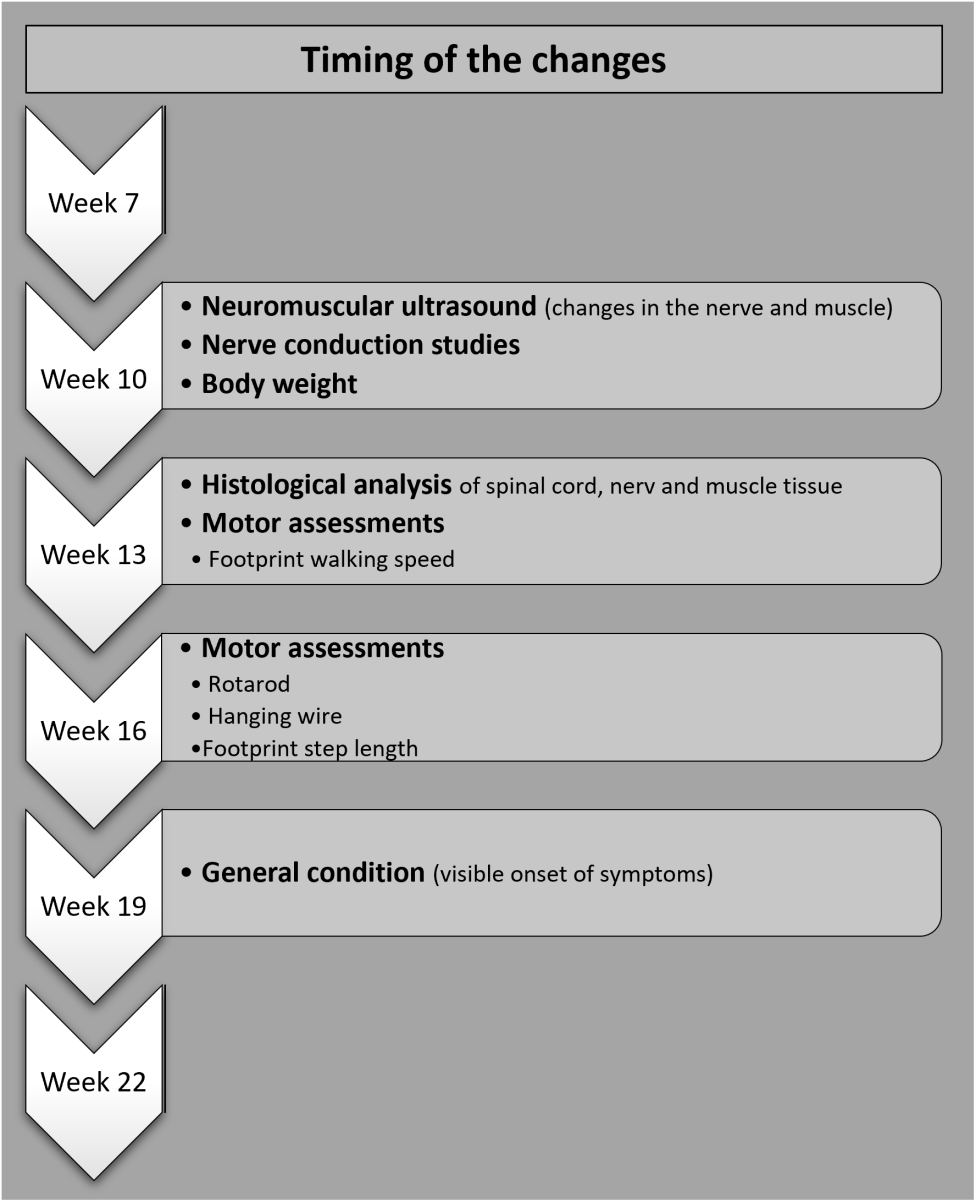
Overview of the timing of disease related changes using different techniques. Detection of significant differences between mutant SOD1^G93A^ and wild-type mice was possible starting from the depicted time points using the different techniques.

Comparing neuromuscular ultrasound to other so far established methods to quantify disease progression, it appeared to be one of the earliest indicators of disease onset in the SOD1^G93A^ mouse model (Fig 9). Already at the age of 10 weeks, first changes in nerve (reduced diameter of the distal sciatic nerve) and muscle (reduced thickness of the gastrocnemius muscle) morphology can be detected by sonography. So far, CMAP amplitudes and body weight have been considered gold standard to detect disease onset in the SOD1^G93A^ mouse model. In our study, a decrease in the CMAP amplitude became evident at the same time as the first changes detectable by ultrasound which might reflect impaired signaling within the altered nerve microstructure. Body weight was also significantly lower compared to wild-type animals at this time point. A possible explanation might be, that the lower body weight results from the loss of muscle tissue, which is corroborated by our sonographic findings. Interestingly, we were only able to detect significant histological changes later, in the cohorts aged 13 weeks and onwards. This might partially be attributed to the fact that we performed histological analyzes on a more proximal level than the ultrasound: number of motor neurons and astrocytosis in the spinal cord, and axon and fiber diameter and distribution in the proximal portion of the sciatic nerve. According to the dying-back hypothesis, those tissues might be affected at a later time point. While we did not see a corresponding decrease of postsynaptic NMJs, an earlier deficit in innervation and earlier loss of the presynapse has previously been reported [71,72].

ROC analysis of the three most promising NMU parameters revealed, that diameter of the distal sciatic nerve as well as echo intensity of the biceps femoris muscle were able to reliably distinguish transgenic from wild-type animals from 7 weeks and 13 weeks onwards, respectively. The CSA of the proximal sciatic nerve only had a limited suitability as a diagnostic tool. However, ROC analysis should be understood as hypothesis generating.

Taken together, our study presents a thorough comparison of invasive and non-invasive techniques and shows for the first time that neuromuscular ultrasound can serve as an early and reliable marker of disease progression in the SOD1^G93A^ mouse model. Future studies will provide further insights into the sequence of and mechanisms behind nerve and muscle degeneration. Given that it is non-invasive and therefore repeatable, neuromuscular ultrasound constitutes an optimal tool to assess the efficacy of new therapeutic strategies in this model of amyotrophic lateral sclerosis. Therefore, including neuromuscular ultrasound is likely to increase the clinical translatability in the future.

## Supporting information

S1 Fig Overview of the set-up of the experiment. (TIF)

S2 Fig Results of the quantitative ultrasound analysis of the right sciatic nerve and hind limb muscles of the transgenic animals over time. (A, B) Representation of the nerve cross-sectional area (CSA) distally (A) and proximally (B) in mutant SOD1G93A mice over time. (C-E) Analysis of nerve diameter in transgenic animals over time. (F-H) Analyses of the gastrocnemius muscle and biceps femoris over time in mutant SOD1G93A mice. Graphs are depicted as interleaved box & whiskers from min. to max. (all data points are shown; the n value is given in the graph). Statistical analysis was performed by using a two-way ANOVA followed by Tukey-Post-hoc-Test. “*” indicates *p* < 0.05; “**” indicates *p* < 0.01; “***” indicates *p* < 0.001.
(TIF)

S3 Fig Results of the quantitative ultrasound analysis of the right sciatic nerve and hind limb muscles of the wild-type animals over time. (A, B) Representation of the nerve cross-sectional area (CSA) distally (A) and proximally (B) in the wild-type control group over time. (C-E) Representation of the analysis of the nerve diameter of wild-type animals over time. (F-H) Analyses of the gastrocnemius muscle and biceps femoris over time in the wild-type control group. Graphs are depicted as interleaved box & whiskers from min. to max. (all data points are shown; the n value is given in the graph). Statistical analysis was performed by using a two-way ANOVA followed by Tukey-Post-hoc-Test. “*” indicates *p* < 0.05; “**” indicates *p* < 0.01; “***” indicates *p* < 0.001.
(TIF)

S4 Fig Correlation analysis of nerve and muscle ultrasound with body weight. The sonographically determined diameter of the distal sciatic nerve and the opening angle of the gastrocnemius muscle do not correlate with body weight. (A, B) The graphs show the correlation of body weight in wild-type animals with the diameter of the distal sciatic nerve (A, Pearson −0.03152, N = 86) and the opening angle of the gastrocnemius muscle (B, Pearson 0.00426, N = 80). (D, E) The graphs show the correlation of the body weight of transgenic animals with the diameter of the distal sciatic nerve (D, Pearson −0.03088, N = 80) and the opening angle of the gastrocnemius muscle (E, Pearson 0.111, N = 76). (C, F) Data from weeks 13–22 are included in the correlation, as a significant increase in body weight was observed in the wild-type (C) and transgenic (F) groups at the first two time points due to growth. Graphs **(A, B, D, E)** are depicted as scatterplot with regression line. (All data points are shown.). Statistical analysis was performed by using Pearson correlation coefficient. Graphs **(C, F)** are depicted as interleaved box & whiskers from min. to max. (all data points are shown). Statistical analysis was performed by using a two-way ANOVA followed by Tukey-Post-hoc-Test. “*” indicates *p* < 0.05; “**” indicates *p* < 0.01; “***” indicates *p* < 0.001.
(TIF)

S5 Data Source data spread sheet. (XLSX)
