# Supplementary figures and images for: Neuromuscular ultrasound as a biomarker in the SOD1 mouse model of amyotrophic lateral sclerosis
Source: PLoS One. 2026 Jul 14;21(7):e0353397. doi: 10.1371/journal.pone.0353397 (PMC13367705; doi:10.1371/journal.pone.0353397)

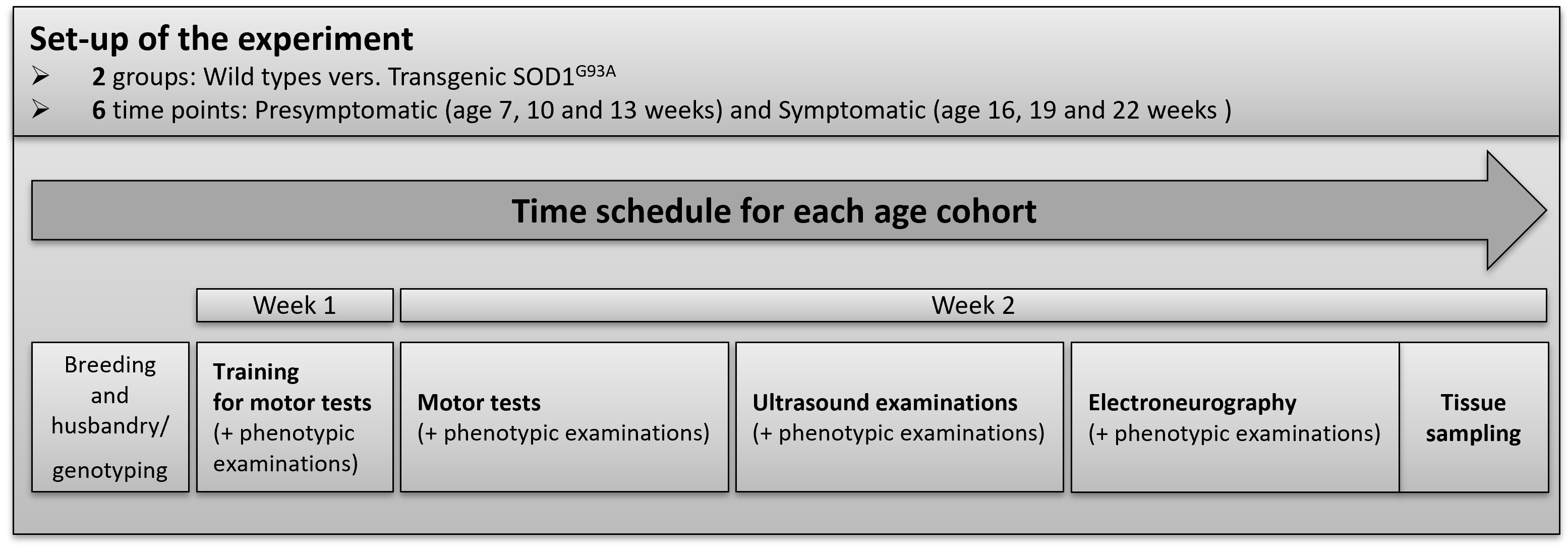

Supplement: S1 Fig — (TIF) [file pone.0353397.s001.tif]

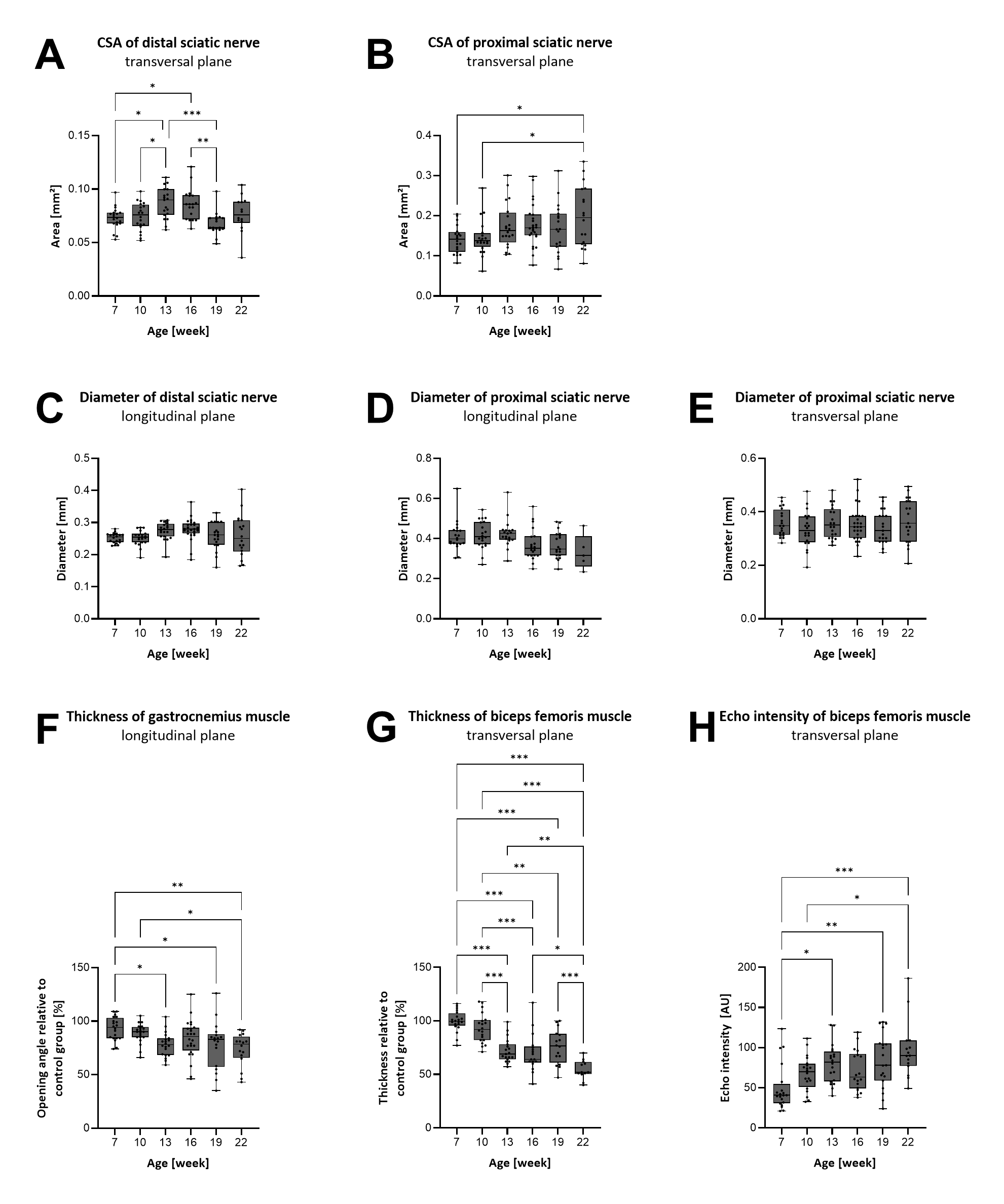

Supplement: S2 Fig — (A, B) Representation of the nerve cross-sectional area (CSA) distally (A) and proximally (B) in mutant SOD1G93A mice over time. (C-E) Analysis of nerve diameter in transgenic animals over time. (F-H) Analyses of the gastrocnemius muscle and biceps femoris over time in mutant SOD1G93A mice. Graphs are depicted as interleaved box & whiskers from min. to max. (all data points are shown; the n value is given in the graph). Statistical analysis was performed by using a two-way ANOVA followed by Tukey-Post-hoc-Test. “*” indicates p < 0.05; “**” indicates p < 0.01; “***” indicates p < 0.001. (TIF) [file pone.0353397.s002.tif]

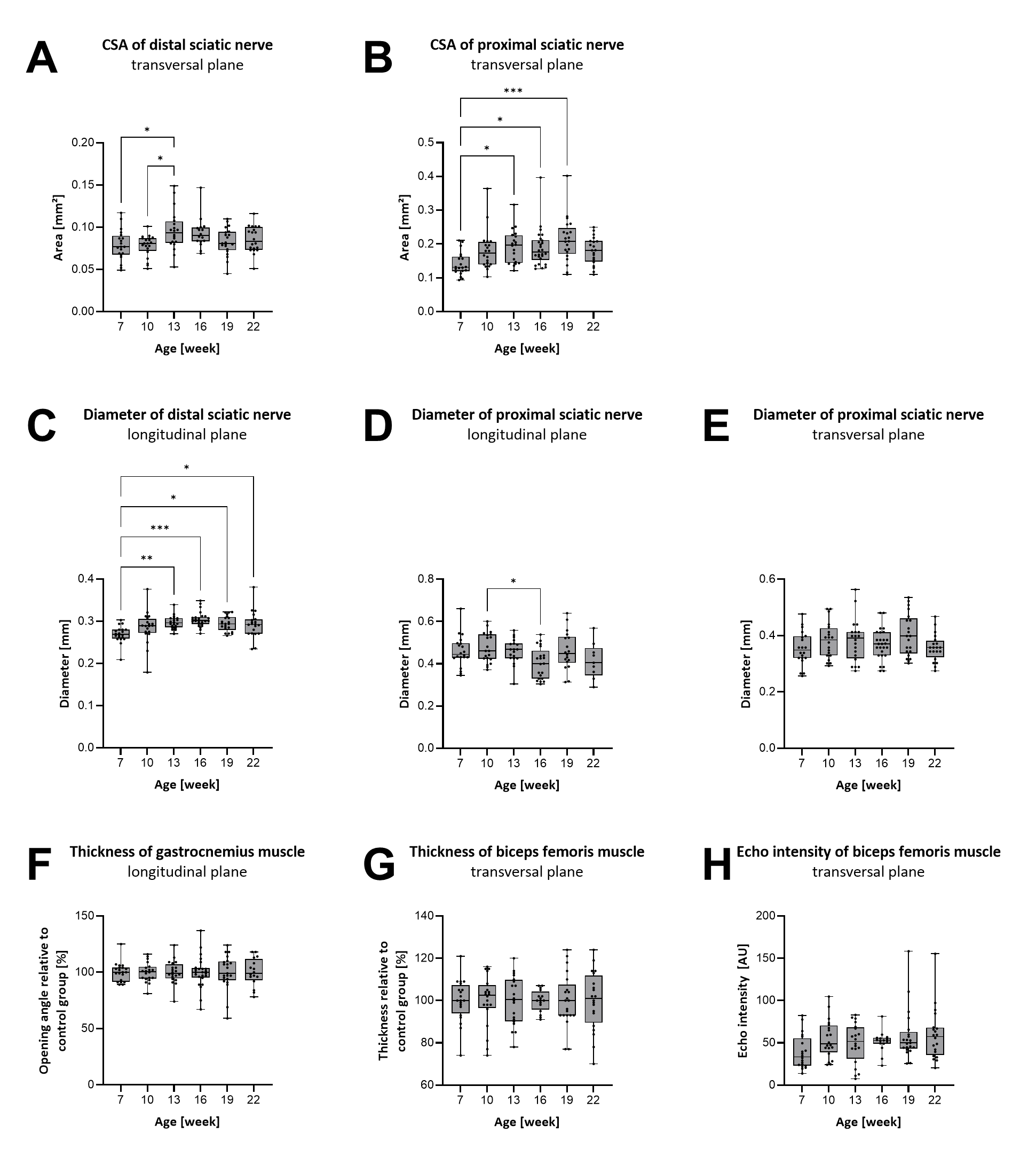

Supplement: S3 Fig — (A, B) Representation of the nerve cross-sectional area (CSA) distally (A) and proximally (B) in the wild-type control group over time. (C-E) Representation of the analysis of the nerve diameter of wild-type animals over time. (F-H) Analyses of the gastrocnemius muscle and biceps femoris over time in the wild-type control group. Graphs are depicted as interleaved box & whiskers from min. to max. (all data points are shown; the n value is given in the graph). Statistical analysis was performed by using a two-way ANOVA followed by Tukey-Post-hoc-Test. “*” indicates p < 0.05; “**” indicates p < 0.01; “***” indicates p < 0.001. (TIF) [file pone.0353397.s003.tif]

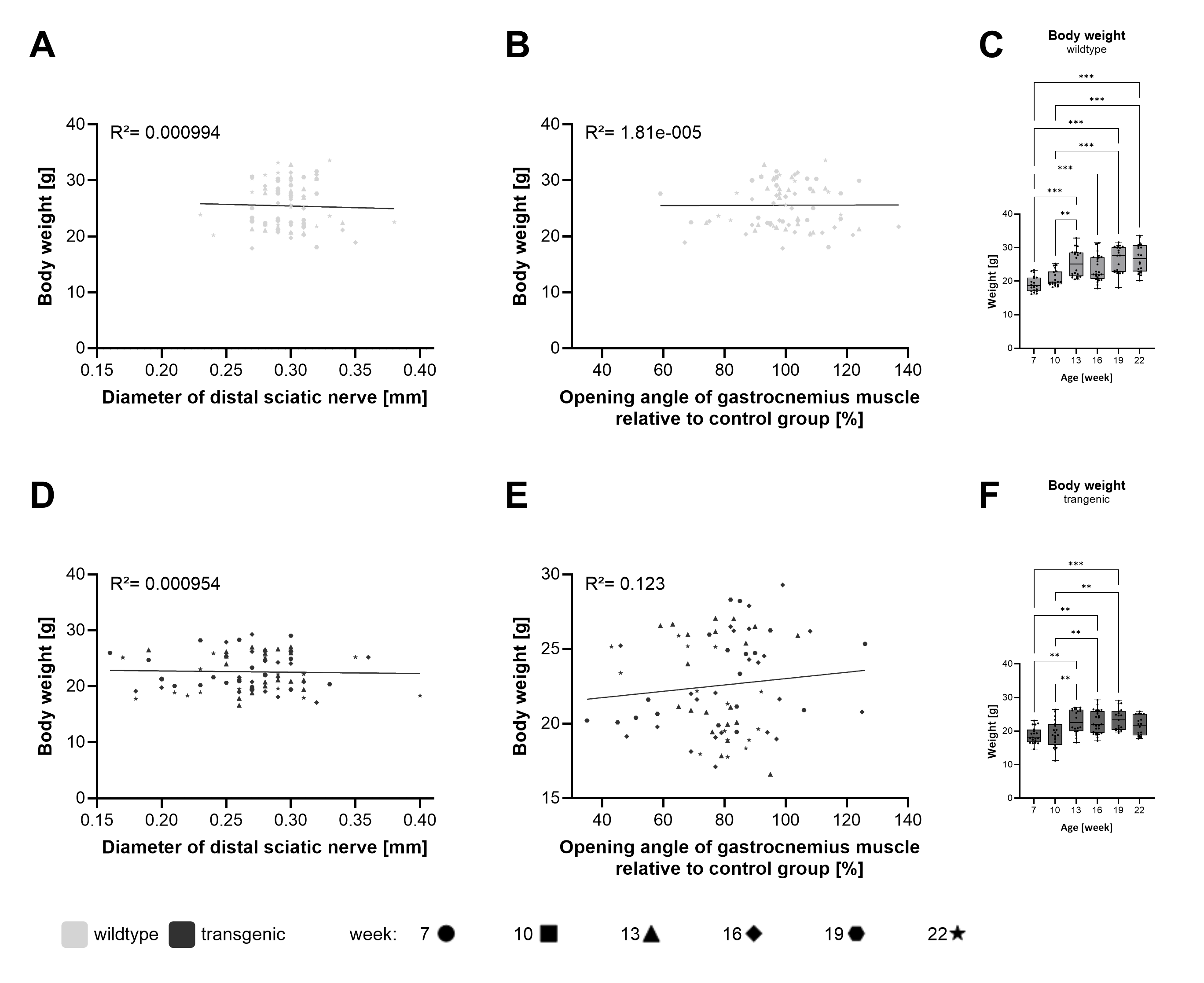

Supplement: S4 Fig — The sonographically determined diameter of the distal sciatic nerve and the opening angle of the gastrocnemius muscle do not correlate with body weight. (A, B) The graphs show the correlation of body weight in wild-type animals with the diameter of the distal sciatic nerve (A, Pearson −0.03152, N = 86) and the opening angle of the gastrocnemius muscle (B, Pearson 0.00426, N = 80). (D, E) The graphs show the correlation of the body weight of transgenic animals with the diameter of the distal sciatic nerve (D, Pearson −0.03088, N = 80) and the opening angle of the gastrocnemius muscle (E, Pearson 0.111, N = 76). (C, F) Data from weeks 13–22 are included in the correlation, as a significant increase in body weight was observed in the wild-type (C) and transgenic (F) groups at the first two time points due to growth. Graphs (A, B, D, E) are depicted as scatterplot with regression line. (All data points are shown.). Statistical analysis was performed by using Pearson correlation coefficient. Graphs (C, F) are depicted as interleaved box & whiskers from min. to max. (all data points are shown). Statistical analysis was performed by using a two-way ANOVA followed by Tukey-Post-hoc-Test. “*” indicates p < 0.05; “**” indicates p < 0.01; “***” indicates p < 0.001. (TIF) [file pone.0353397.s004.tif]
